# APELLA: Open-Source, miniaturized All-in-One powered Lab-on-a-Disc platform

**DOI:** 10.1016/j.ohx.2023.e00449

**Published:** 2023-06-27

**Authors:** Laura Serioli, Atsushi Ishimoto, Akinobu Yamaguchi, Kinga Zór, Anja Boisen, En-Te Hwu

**Affiliations:** a The Danish National Research Foundation and Villum Foundation’s Center for Intelligent Drug Delivery and Sensing Using Microcontainers and Nanomechanics (IDUN), Department of Health Technology, Technical University of Denmark, Lyngby, Denmark; b Laboratory of Advanced Science and Technology for Industry (LASTI), University of Hyogo, Japan; c BioInnovation Institute Foundation, Copenhagen N 2800, Denmark

**Keywords:** Arduino, Real-time imaging, Propellers, Centrifugal microfluidics, Wireless power, Lab-on-a-Disc

## Abstract

We present an unconventional approach to a common Lab-on-a-Disc (LoD) that combines a quadcopter propulsion system, a miniaturized 2.4 GHz Wi-Fi spy camera, 9.74 Watt Qi wireless power, and an Arduino into an open-source, miniaturized All-in-one powered lab-on-disc platform (APELLA). The quadcopter propulsion generates thrust to rotate (from 0.1 to 24.5 Hz) or shake the LoD device, while the spy camera enables a real-time (30 frames per second) and high definition (1280 × 720 pixels) visualization of microfluidic channels without requiring a bulky and heavy stroboscopic imaging setup. A mobile device can communicate with an Arduino microcontroller inside the APELLA through a Bluetooth interface for closed loop and sequential frequency control. In a proof-of-concept study, the APELLA achieved comparable mixing efficiency to a traditional spin stand and can capture rapid microfluidic events at low rotational frequencies (<5Hz). The APELLA is low-cost (c.a. 100 Euro), compact (15.6 × 15.6 × 10 cm^3^), lightweight (0.59 kg), portable (powered by a 5 V USB power bank), and energy efficient (uses < 6% power of the conventional system), making it ideal for field deployment, education, resource-limited labs.

## Graphical abstract

**Figure:**
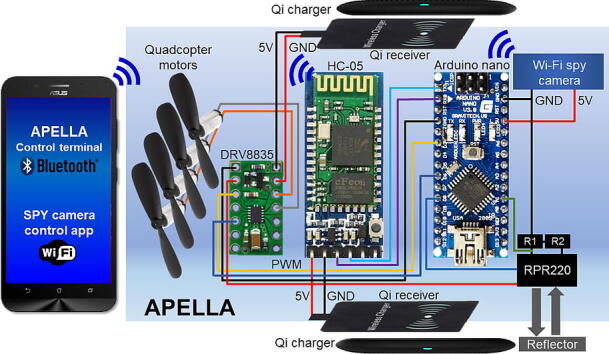

## Specifications table

**Table:** 

Hardware name	All-in-one PowEred Lab-on-a-Disc pLAtform (APELLA)
Subject area	• Open-source alternatives to existing infrastructure • Microfluidics • Engineering and materials science • Chemistry and Biochemistry • Biological Sciences • Educational tools • General
Hardware type	• Imaging tools • Measuring physical properties and in-lab sensors • Biological sample handling and preparation • Field measurements and sensors • Electrical engineering and computer science • Mechanical engineering and materials science
Closest commercial analog	Conventional spin-stand with stroboscopic imaging system
Open-source license	CC BY-SA 4.0
Cost of hardware	EUR 109
Source file repository	https://doi.org/10.17605/OSF.IO/HB6UV

## 1 Hardware in context

Centrifugal microfluidic platforms, or Lab-on-a-Disc (LoD), exploit the centrifugal force to actuate and move liquids on a disc-shaped device, allowing the implementation of different operational units such as metering, valving, mixing, and centrifugation [1], [2], [3], [4]. The main advantage of the LoD platforms compared to Lab-on-a-Chip setups is the significant reduction of dead volumes [5] since the LoDs do not require additional pumps or tubing. This leads to the reduction of reagent volumes as well as bubble formation [6], [7]. In LoDs, multiplexed complex assays can be performed rapidly and reliably [8]. Moreover, the disposable disc can be mass-produced, reducing the risk of sample cross-contamination. Thus, LoDs have been used for point-of-care testing [6], [9], biochemical assays [10], environmental analysis [4], [10], and cell-based assays [11], [12].

**Fig. 1:**
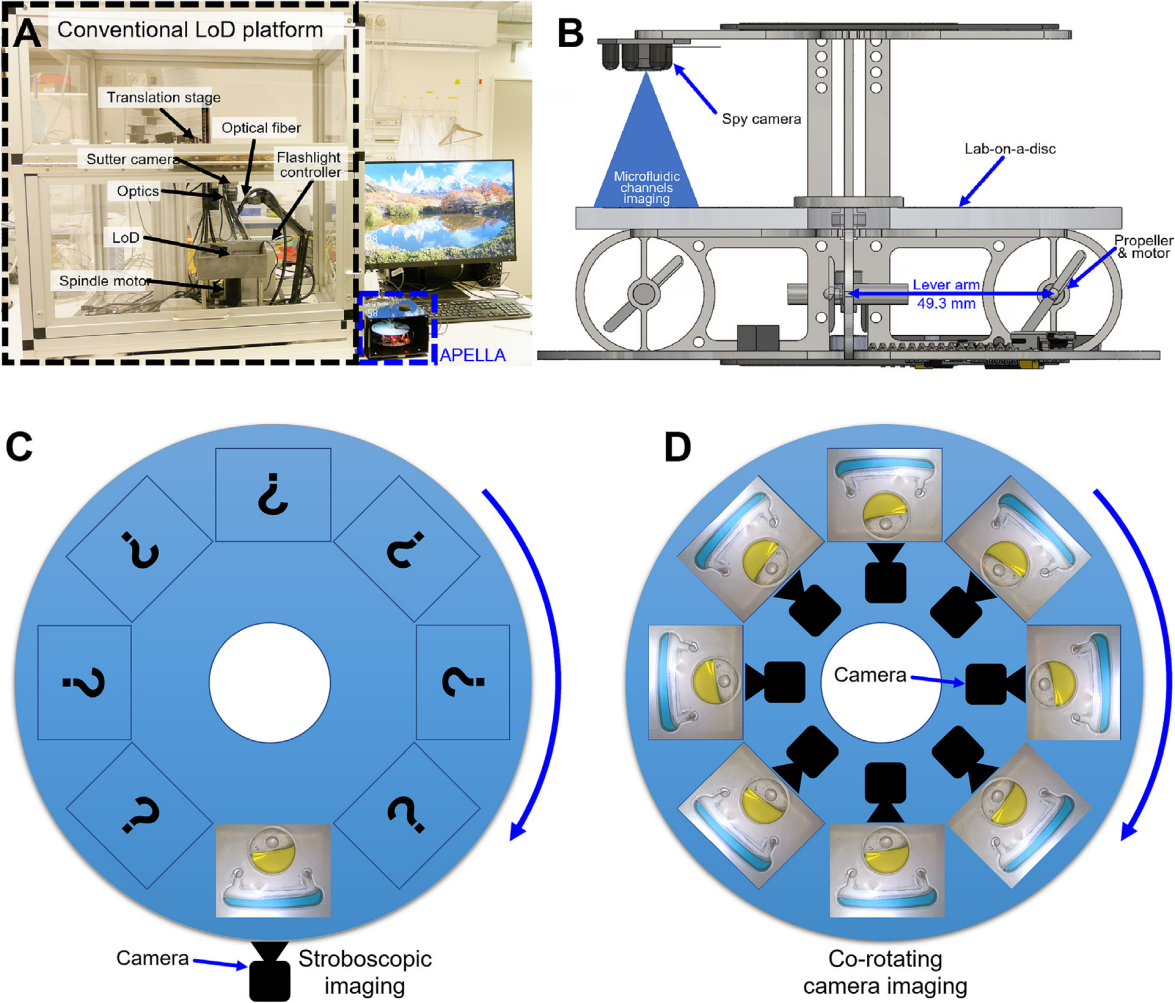
Comparison between a conventional stroboscopic imaging setup and the APELLA. **A)** Size comparison of two platforms. **B)** APELLA implements high efficiency and light weight motor-propeller based driving mechanism with a 49.3 mm long lever arm to drive the rotational part. The APELLA integrates a miniaturized spy camera on top of a microfluidic disc for co-rotating camera imaging. The spy camera provides a fixed temporal resolution (30 frames per second) unrelated to the rotation speed. **C)** The stroboscopic system takes one high-resolution image of microscale channels per revolution. **D)** The spy camera follows and monitors the channels on the disc.

Despite the DVD size, LoDs need auxiliary equipment to function. The liquid actuation requires an energy-consuming spindle motor (such as Maxon RE35). Additionally, there is a need for equipment such as a potentiostat [13], [14], spectrophotometer [15], and optical microscope [16] for detection and monitoring. Moreover, real-time optical imaging is essential when following biological events [17], [18], chemical reactions [18] and physical events such as release [19] and mixing [20], [21]. LoD platforms commonly use a rather bulky and complex strobscopic imaging setup (such as Polytec BVS-II Wotan) to monitor liquid movement on the disc in real time. The stroboscopic setup needs synchronized flash light triggering, microscopic optics and a shutter camera (such as pco.pixelfly USB with 1392 × 1040 pixels), as shown in Fig. 1A [22], [23]. Traditional stroboscopic setup takes one high-resolution image per revolution, making rapidly changing events hard to monitor on the disc at other rotational angles, as shown in Fig. 1C. Although commercial instruments exist where the optical detection and the spin stand are integrated [24], the usage of optical detection during rotation is rather challenging [25]. Furthermore, for time/environment-sensitive experiments, stopping and moving the disc may increase unwanted uncertainty that interferes with the experiment results.

Recent studies have shown how wireless power transmission can be used to power active electrical components on a rotating disc [26], [27], [28], which opens up a myriad of possibilities for on-disc applications such as actuation [29], [30], electrochemical [19], [31], chemiluminescence [8], microscopic imaging [32], [33], and cell culture monitoring [34]. The development of wireless-powered LoD systems shows the potential of this platform to be used for on-site detection, considering the substantial reduction of auxiliary instrumentation [8]. In order to increase the degree of portability and the possibility for point-of-need real-time monitoring, various detection units have been specially designed and integrated with LoD platforms [33].

This work presents an unusual approach that integrates an energy-efficient quadcopter propulsion mechanism, a miniature Wi-Fi spy camera, and Qi wireless power into an open-source and miniaturized APELLA for LoD applications, as shown in Fig. 1A. To the best of our knowledge, APELLA is the first LoD platform that integrates a propeller driving mechanism. The propeller-driven mechanism was chosen because the rotational part (including camera, LoD, framework, and electronics) weighs 96.3 g and require a spindle motor with high enough torque to rotate. However, such a motor is heavy (>100 g) and needs an input voltage of at least 24 V or higher. To keep the LoD/imaging system lightweight, portable, easy to control (single Arduino directly controls motors, sensors and actuators on the disc), and easy to power (5 V power bank), the light-weight motor-propeller (total c.a. 12 g for 4 units) driving mechanism seems to be an optimal solution. This is because the distance between the propeller and the rotation axis is 49.3 mm, which acts as a long lever arm (as shown in Fig. 1B) to increase the torque required to drive the rotational part. The spy camera can provide real-time optical monitoring with an imaging temporal resolution decoupled from the rotating speed (Fig. 1D). Furthermore, the spy camera can monitor rapid events with a 33-millisecond interval. The goal of APELLA is to simplify the engineering complexity of the LoD setup, enabling individuals without a professional engineering background to build and conduct LoD research and applications with ease.

## 2 Hardware description

**Fig. 2:**
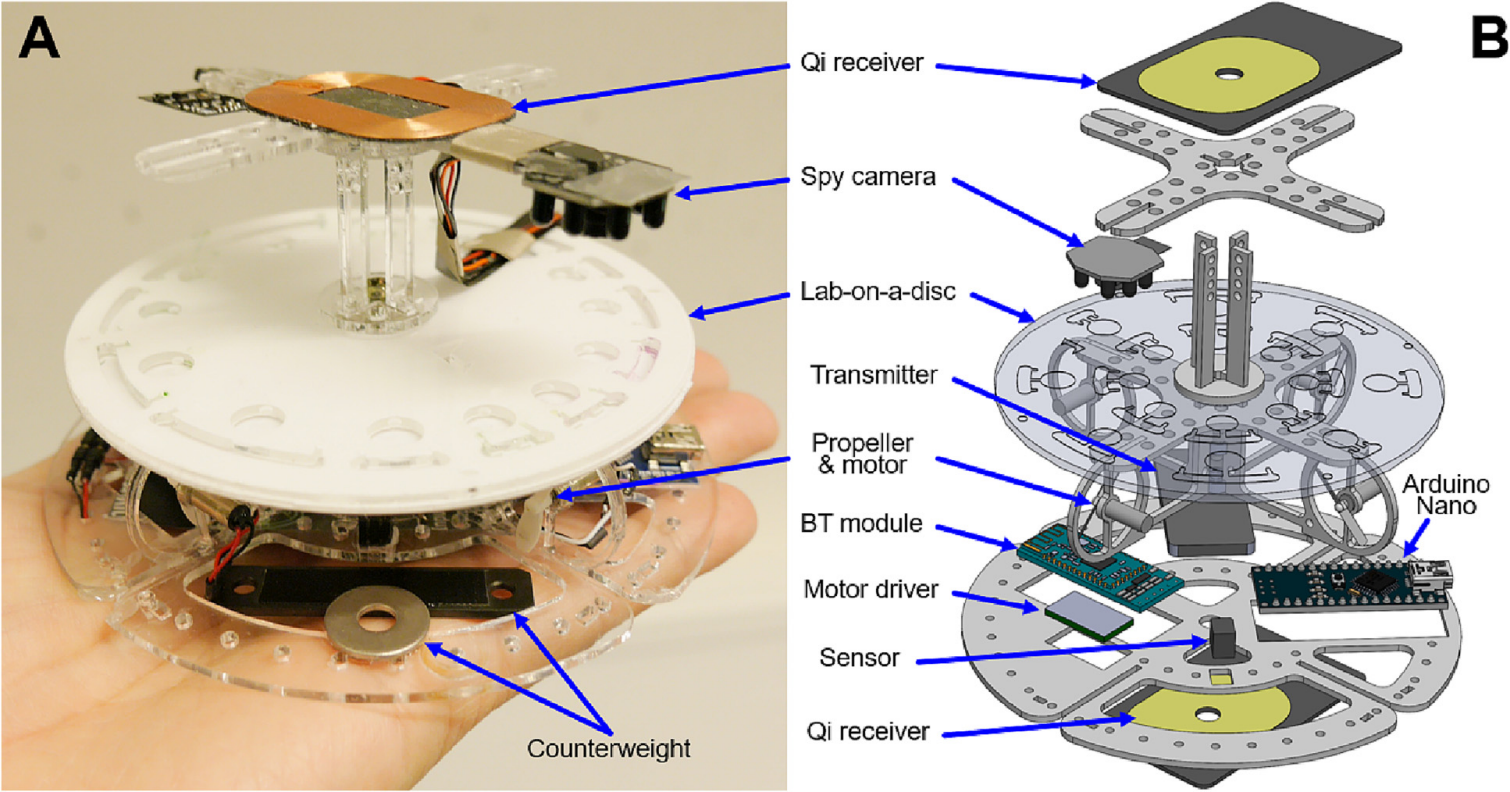
Photo and exploded diagram of a rotational part of the APELLA. **A)** The rotational part weighs 96.3 g and has a similar size to a palm. **B)** This part contains two Qi power receivers, Wi-Fi/Bluetooth signal transmitters, a spy camera, a proposition mechanism, an optical sensor, and an Arduino Nano microcontroller.

APELLA is a hand-held sized device built with low-cost and off-the-shelf electronics such as Arduino nano (Arduino, Italy), Bluetooth module (HC-05, Ziihao, China), optical sensor (RPR220, Rohm, Japan), motor driver board (DRV8835), mobile phone Qi [35] wireless inductive chargers (bxcqi01, KSIX, Spain), receivers (SmartTec, China), miniature Wi-Fi spy camera (ULar, China) and four lightweight and high-efficiency motor-propeller units from a mini-quadcopter (YuuHeeER, China). Rather than utilizing a metallic material, the APELLA framework is constructed from lightweight polymethyl methacrylate (PMMA), measuring at a thickness of 1.6 mm. The PMMA not only reduces rotational inertia but is also easy to machine. Fig. 2 shows a photo (Fig. 2A) and an exploded diagram of components (Fig. 2B) in a rotational part of the APELLA. The bottom Qi receiver powers the Arduino nano microcontroller, the Bluetooth 2.4 GHz transmitter module, electronic modules, and the Wi-Fi spy camera. Another Qi receiver on top of the framework powers the motor driver board to drive the motor-propeller based propulsion mechanism. The Arduino reads the rotational speed signal from the reflective optical sensor and sends a pulse width modulation (PWM) signal to the motor driver for rotation speed control. The spy camera is fixed on top of the PMMA framework and focused on the LoD for microfluidic channel monitoring.

The APELLA was characterized to see how it could be used by studying the mixing process of two colored solutions in a LoD. Additionally, the mixing performance of the APELLA was compared with that of a conventional spin stand while using identical parameters. The spy camera was used to observe the mixing solution injection event with a high imaging temporal resolution.

In summary, the presented APELLA provides:

• A cost-effective and open-source platform for LoD research and applications.
• High imaging temporal resolution regardless of rotation speed.
• Wireless power for support of imaging and other electronic sensors on the disc.
• Single Arduino microcontroller for total system control.
• No need for customized printed circuit boards and metal machining parts.

## 3 Design files

The APELLA parts were designed using SolidWorks 2022 (Dassault Systèmes, Vélizy-Villacoublay, France) computer-aided design (CAD) software. All the design files are available in IGES format and can be downloaded from the linked Open Science Framework (OSF) file repository.

## 3.1 Design files summary

**Table:** 

**Design file name**	**File type**	**Open source license**	**Location of the file**
P1.iges	CAD	CC BY-SA 4.0	https://doi.org/10.17605/OSF.IO/HB6UV
P2.iges	CAD	CC BY-SA 4.0	https://doi.org/10.17605/OSF.IO/HB6UV
P3.iges	CAD	CC BY-SA 4.0	https://doi.org/10.17605/OSF.IO/HB6UV
P4.iges	CAD	CC BY-SA 4.0	https://doi.org/10.17605/OSF.IO/HB6UV
P5.iges	CAD	CC BY-SA 4.0	https://doi.org/10.17605/OSF.IO/HB6UV
P6.iges	CAD	CC BY-SA 4.0	https://doi.org/10.17605/OSF.IO/HB6UV
P7.iges	CAD	CC BY-SA 4.0	https://doi.org/10.17605/OSF.IO/HB6UV
P8.iges	CAD	CC BY-SA 4.0	https://doi.org/10.17605/OSF.IO/HB6UV
P9.iges	CAD	CC BY-SA 4.0	https://doi.org/10.17605/OSF.IO/HB6UV
APELLA.iges	CAD	CC BY-SA 4.0	https://doi.org/10.17605/OSF.IO/HB6UV

### 3.2 Cutting files

All the laser-cutting files are in DXF format and available on the OSF file repository. P1-P6 (1.6 mm thickness), D1, D2, D4 (2 mm thickness), and D3 (0.5 mm thickness) were cut with optical grade transparent PMMA plates (Goodfellow, Cambridge, England). P7-P9 (3 mm thickness) were cut with black PMMA plates. A tabletop laser cutter (Epilog Mini 18, Epilog Laser, Golden, CO, USA) was used with a speed, laser power, and frequency setting of 10–40 % and 5,000 Hz, respectively. T1-T3 are 84 µm thick double-sided-pressure-sensitive adhesive (PSA) tape (ARcare 7840, Adhesive Research, Limerick, Ireland) which were cut using a precision blade cutter (CE-40, Graphtech, U.S.A.).

**Table:** 

**Design file name**	**Number parts**	**Open source license**	**Location of the file**
P1.dxf	2	CC BY-SA 4.0	https://doi.org/10.17605/OSF.IO/HB6UV
P2.dxf	1	CC BY-SA 4.0	https://doi.org/10.17605/OSF.IO/HB6UV
P3.dxf	1	CC BY-SA 4.0	https://doi.org/10.17605/OSF.IO/HB6UV
P4.dxf	1	CC BY-SA 4.0	https://doi.org/10.17605/OSF.IO/HB6UV
P5.dxf	1	CC BY-SA 4.0	https://doi.org/10.17605/OSF.IO/HB6UV
P6.dxf	1	CC BY-SA 4.0	https://doi.org/10.17605/OSF.IO/HB6UV
P7.dxf	2	CC BY-SA 4.0	https://doi.org/10.17605/OSF.IO/HB6UV
P8.dxf	2	CC BY-SA 4.0	https://doi.org/10.17605/OSF.IO/HB6UV
P9.dxf	2	CC BY-SA 4.0	https://doi.org/10.17605/OSF.IO/HB6UV
D1.dxf	1	CC BY-SA 4.0	https://doi.org/10.17605/OSF.IO/HB6UV
D2.dxf	1	CC BY-SA 4.0	https://doi.org/10.17605/OSF.IO/HB6UV
D3.dxf	1	CC BY-SA 4.0	https://doi.org/10.17605/OSF.IO/HB6UV
D4.dxf	1	CC BY-SA 4.0	https://doi.org/10.17605/OSF.IO/HB6UV
T1.dxf	1	CC BY-SA 4.0	https://doi.org/10.17605/OSF.IO/HB6UV
T2.dxf	1	CC BY-SA 4.0	https://doi.org/10.17605/OSF.IO/HB6UV
T3.dxf	1	CC BY-SA 4.0	https://doi.org/10.17605/OSF.IO/HB6UV

### 3.3 Arduino code

The Arduino code was programmed with an Arduino integrated development environment. The Arduino code references a tachometer sketch (courtesy of the author is in the code) and a proportional-integral-derivative (PID) controller (PID_v1.h from Arduino library) that provide precise rotation speed and PID motor control. The PID controller has two stages, where we apply the first stage PID (aggKp, aggKi, and aggKd) when the rotational speed deviates significantly from a speed setpoint. Conversely, we use the second stage PID (consKp, consKi, and consKd) when the rotational speed is close to the setpoint. To prevent overshoot when changing rotation speed, it is needed to fine tune the PID parameters based on the rotation momentum of the LoD. Moreover, the code facilitates the communication between the Bluetooth module and mobile devices for speed parameter setting. Furthermore, the code generates a pulse width modulation (PWM) signal to the motor driver (DRV8830) that drives four motors for the APELLA propulsion. Changing the PWM frequency from 490 to 31,372.55 Hz is essential to avoid abnormal current consumption of the DRV8830 while driving the motors. When uploading a modified Arduino code, removing the Qi receiver ground and power from the Arduino Nano board is suggested.

### 3.4 Electronics

**Fig. 3:**
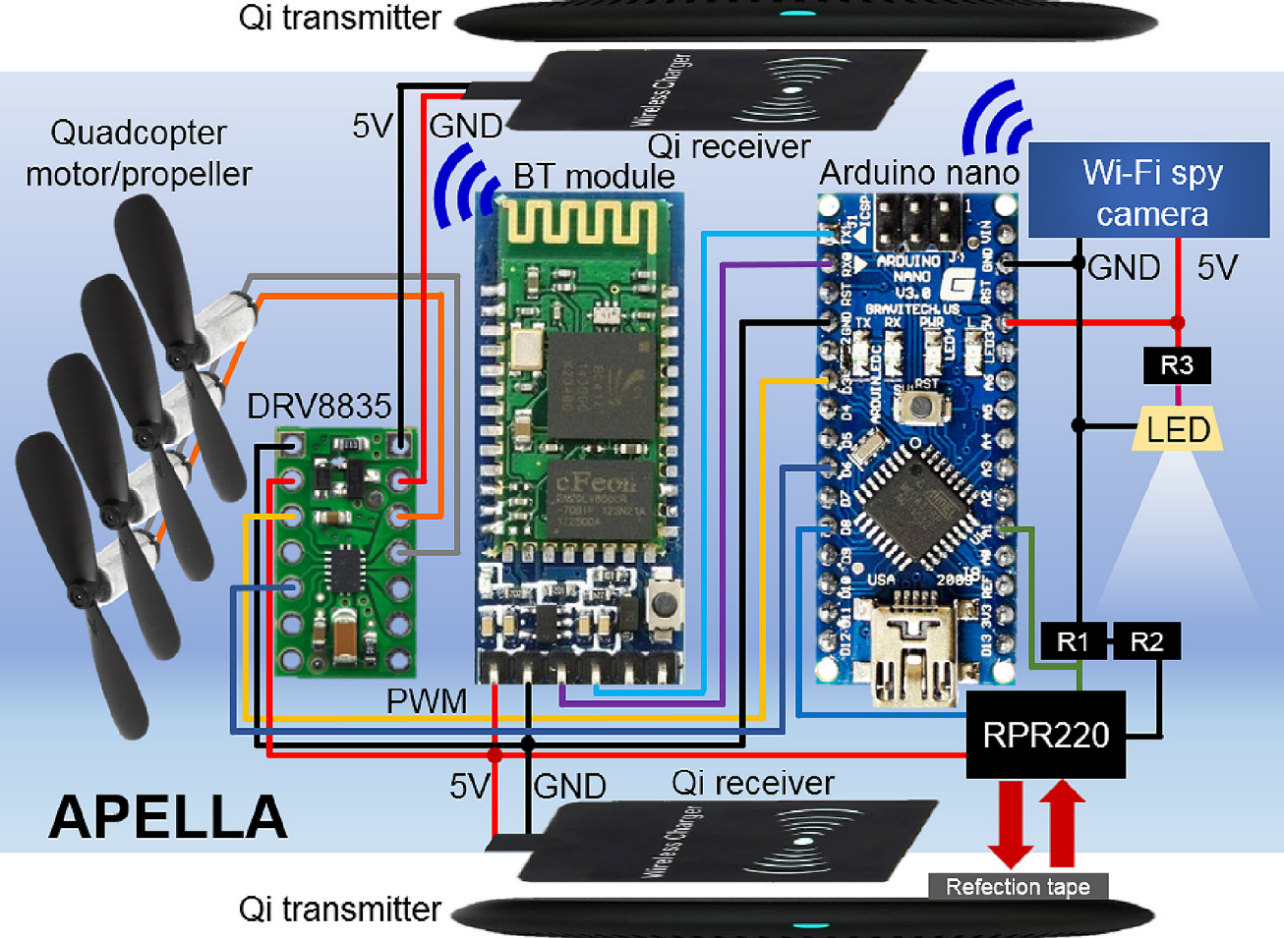
The electrical connection of the APELLA. There is no need for custom-made printed circuit boards, which simplifies the development process. Where the R1, R2 and R3 are 68 k, 200 and 1 k Ohm, respectively.

Two 205 kHz Qi wireless transmitters transmit 5 V and 0.96 A power via bottom and top Qi receivers to the rotation part of APELLA. A detailed circuit connection is shown in Fig. 3. The bottom Qi receiver powers the Arduino nano, the Bluetooth 2.4 GHz module, the Wi-Fi spy camera controller, and the motor driver. The RPR220 optical sensor provides a zero-point (when passing a reflection tape) signal to the Arduino connected to the motor driver. The top Qi receiver is connected to the input of the motor driver, which has a parallel connection to four motors. The maximum power consumption of APELLA is 9.74 W, <6% of the power consumption in a conventional spin stand (such as Maxon RE35, 90 W) with a stroboscopic imaging system (Polytec BVS-II Wotan, 75 W).

## 4 Bill of materials summary

In addition to the cutting components, items were purchased from other sources. An overview of these components, the number required, as well as the cost can be found in the table below.

**Table:** 

**Designator**	**Component**	**Number**	**Cost per unit- €**	**Total cost- €**	**Source of materials**	**Material type**
*Motor-propeller*	*Disassembled from a mini drone (YuuHeeER)*	4	5	20	Amazon	Metal
Arduino	Arduino Nano	1	7.5	7.5	Amazon	Electronics
BT module	HC-05 Bluetooth module	1	3.6	3.6	Amazon	Electronics
Infrared sensor	RPR 220	1	1.6	1.6	Digi-key	Electronics
Motor driver	DRV8835	1	10.3	10.3	RobotShop	Electronics
Qi charger	KSIX bxcqi01 charger with central aperture.	2	19.9	39.8	Amazon	Electronics
Qi receiver	Universal Qi receiver.(1,000 mA, 5 V)	2	1.8	3.6	Amazon	Electronics
Spy camera & transmitter	Miniature Wi-Fi Spy camera	1	17	17	Amazon	Electronics
LED	White light LED	1	0.66	0.66	Digi-key	
Bearing	NMB ball bearing 730ZZHA1P25LY121	2	1.8	3.6	RS	Metal
Rubber foot	BS17BL06X12RP	4	0.17	0.68	Digi-key	Rubber
M3 × 5 screw	M3 × 8 mm Hex head cap screw	1	0.05	0.27	Misumi	Metal
M3 × 8 screw	M3 × 8 mm Hex head cap screw	5	0.03	0.27	Misumi	Metal
M3 × 30 screw	M3 × 30 mm Hex head cap screw	1	0.03	0.03	Misumi	Metal
M3 nut	M3 hex nut	7	0.02	0.14	Misumi	Metal

## 5 Build instructions

**Fig. 4:**
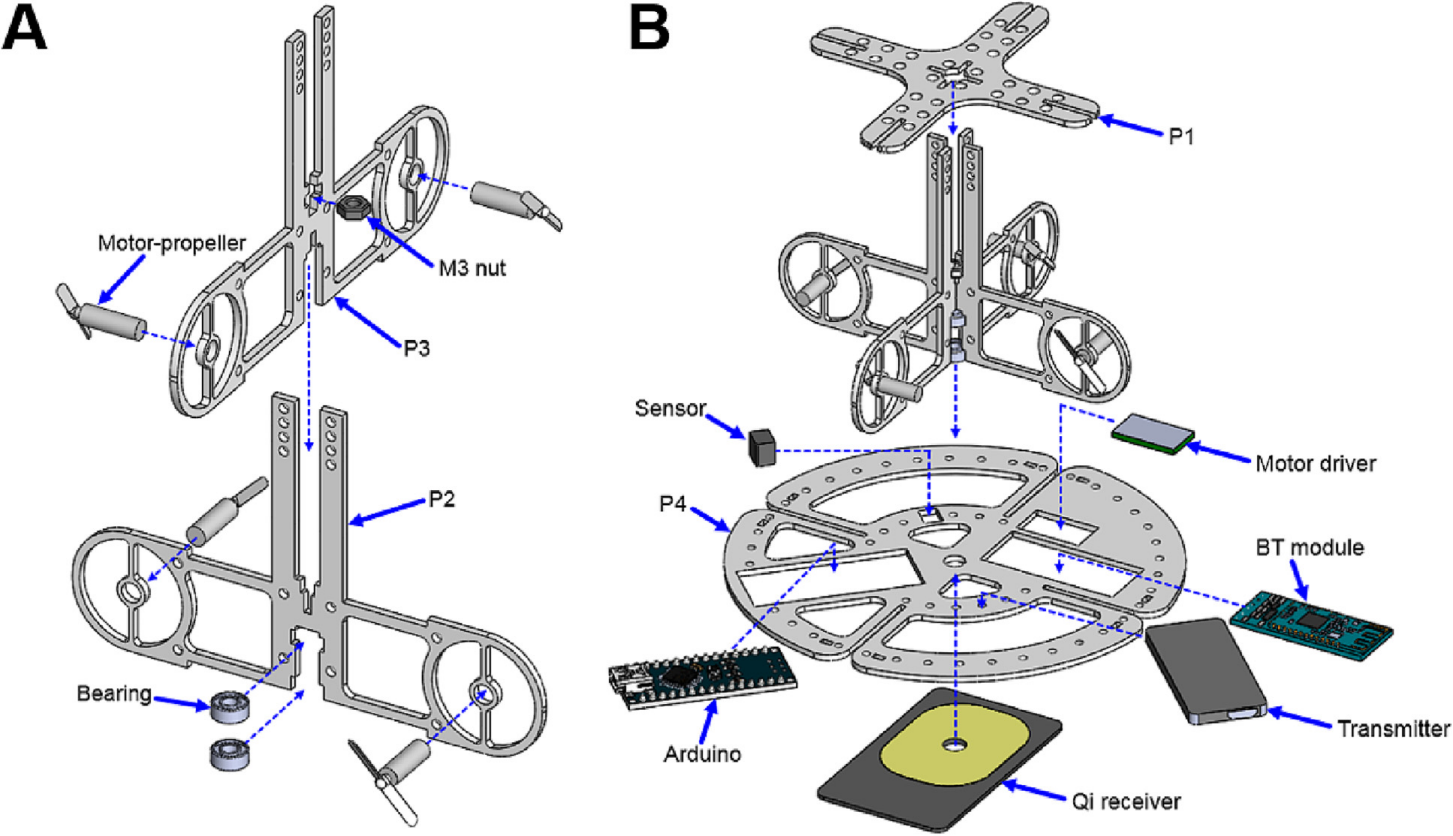
Framework assembly: **A)** propeller mechanism and **B)** system framework.

**Fig. 5:**
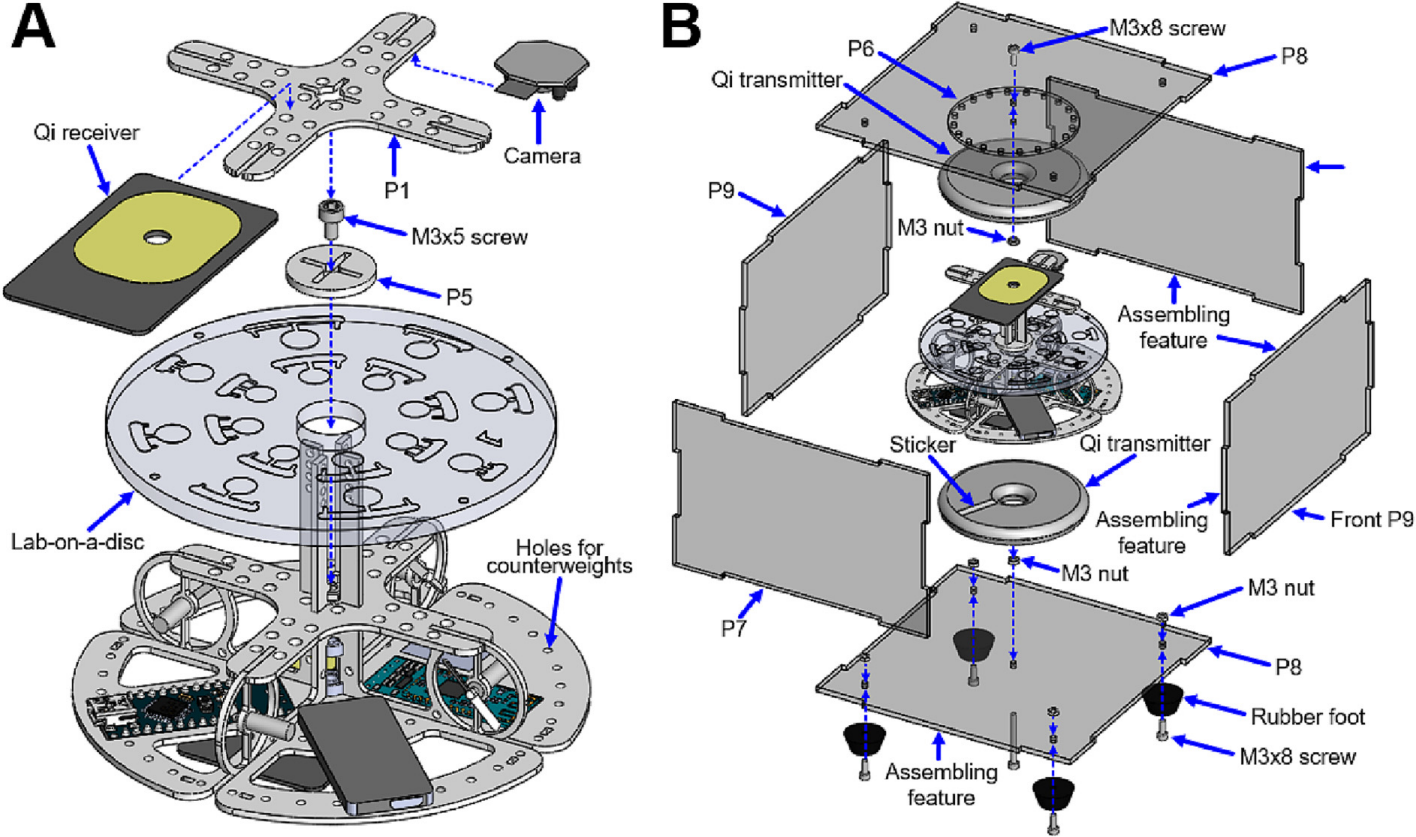
APELLA assembly: **A)** imaging system and **B)** black PMMA housing.

**Fig. 6:**
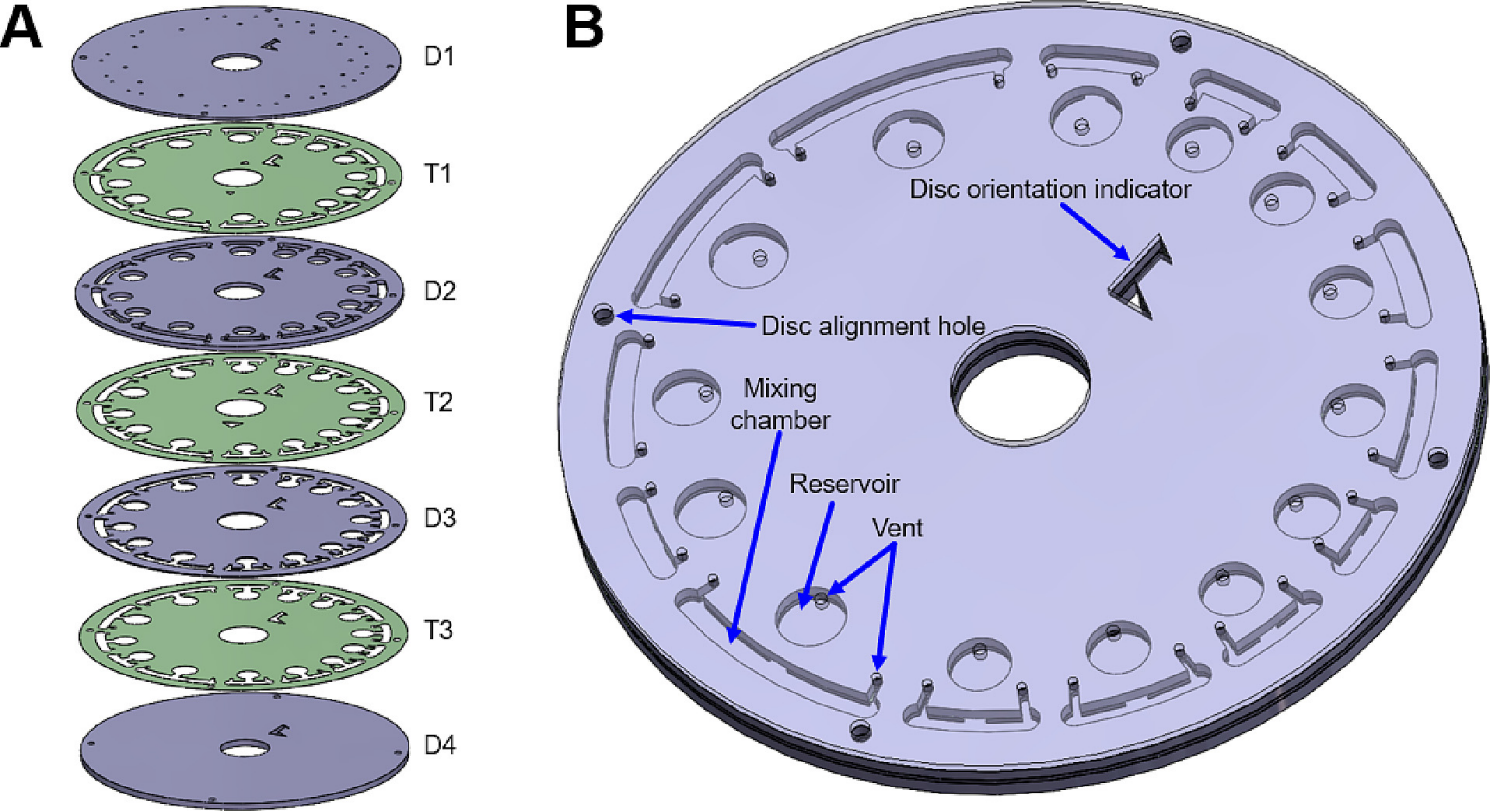
Mixing LoD with four (10, 15, 20, 25 mm) different lengths of the bottom reservoir design. **A)** Exploded view of the mixing LoD with different PMMA and PSA layers and **B)** assembled mixing LoD with inlets, top reservoirs, and different size bottom reservoirs. The channels that connect the reservoir to the mixing chamber are fabricated in T2, D3, and T3.

The assembly requires instant glue (Loctite Super Liquid 20 GR, Henkel, Düsseldorf, Germany) to ensure firm bonding between the components. A roller hand tool is needed for the mixing LoD assembly. Fig. 4, Fig. 5, Fig. 6.

### 5.1 Propeller mechanism assembly (Fig. 4A)

1. Components needed: 4 × motor-propellers, 2 × bearings, 1 × P2, 1 × P3 and 1 × M3 nut.
2. Insert and glue four motor-propeller into holes on the P2 and P3.
3. Insert the M3 nut into a central feature on the P3.
4. Insert the bearings into a bottom feature on the P2.
5. Slide the P3 into the central concave feature of the P2.
6. Glue the M3 nut to the P2 and P3.
7. Glue the outer ring of the bearings to the P2 and P3 (notice: make sure the glue is not gluing the inner rotatable ring of the bearings).

### 5.2 System framework assembly (Fig. 4B)

1. Components needed: 1 × propeller mechanism assembly, 1 × P1, 1 × P4, 1 × Arduino, 1 × Qi receiver, 1 × transmitter, 1 × BT module, 1 × motor driver and 1 × sensor.
2. Drill a hole at the center of the Qi receiver coil.
3. Glue the Arduino, transmitter, BT module, motor driver, sensor, and Qi receiver to the P4.
4. Insert and glue the P1 onto the propeller mechanism assembly.
5. Glue the propeller mechanism assembly to the P4.
6. Connect electric wires between the motors and the circuits.

### 5.3 Imaging system assembly (Fig. 5A)

1. Components needed: 1 × system framework assembly, 1 × LED, 1 × P1, 1 × P5, 1 × spy camera, 1 × Qi receiver, 1 × M3 × 5 screw and 1x LoD.
2. Insert the LoD and the P5 into the system framework assembly.
3. Fix the P5 and the LoD with the M3 × 5 screw (if the disc is thicker, use M3x8 or longer screw)
4. Drill a hole at the center of the Qi receiver coil.
5. Align the hole of the Qi receiver to the center of the P1.
6. Glue the Qi receiver to the P1.
7. Insert a cross feature on top of the system framework assembly to a central cross-shaped hole of the P1.
8. Glue the LED to the camera.
9. Connect the camera to the receiver and adjust the camera focus and imaging area (field of view) on the disc.
10. Glue the camera to the bottom of the P1.
11. Fix screws and nuts as counterweights into holes surrounding the P4 for balanced weight distribution during rotation.

### 5.4 Housing assembly (Fig. 5B)

1. Components needed: 5 × M3 × 8 screws, 1 × M3 × 30 screw, 6 × M3 nuts, 4 × rubber foots, 2 × P7, 2 × P8, 2 × P9, 2 × Qi transmitters, 1 × reflective sticker.
2. Assemble the rubber feet with M3 × 8 screws and M3 nuts on the lower P8.
3. Insert and fix the M3 × 30 screw to a central hole of the lower P8 with the M3 nut.
4. Align the Qi transmitter center to the M3 × 30 screw.
5. Glue the Qi transmitter to the lower P8.
6. Place the reflective sticker (aluminum or copper tape) on the Qi transmitter.
7. Fix the P6 with the M3 × 8 screw and M3 nut on the upper P8.
8. Align the Qi transmitter center to the M3x8 screw.
9. Glue the Qi transmitter to the P6.
10. Align the assembling features, glue left/right P7 and back P9 to the upper P8 as upper housing.
11. Insert the M3 × 30 screw into the bearings of the framework assembly.
12. Align the upper P8 assembly to the lower P8 assembly.
13. Assemble the front P9 (without glue)

### 5.5 Mixing LoD assembly (Fig. 6A-B)

1. Components needed: 1 × D1-D4 PMMA parts and 1 × T1-T3 PSA tapes.
2. Insert 3 mm diameter alignment pins on D4 and place T3 on top of D4.
3. Place D3 on top of T3 and use a roller to press D3 and D4.
4. Place T2 on top of D3, then D2 on top of T2.
5. Use the roller to press D2.
6. Place T1 on top of D2, then D1 on top of T1.
7. User roller to press D1.

## 6 Operation instructions

1. Install P2PCAMAP spy camera (https://apkfab.com/p2pcamap/com.g_zhang.P2PCAMAP) and Serial Bluetooth Terminal apps (https://m.apkpure.com/serial-bluetooth-terminal/de.kai_morich.serial_bluetooth_terminal) to a mobile device to control APELLA.
2. Connect the top and bottom Qi transmitter to the 5 V USB power source.
3. Pair the BT module with a mobile device.
4. Connect the mobile device to the spy camera Wi-Fi hotspot.
5. Launch the P2PCAMAP app, find and add the spy camera.
6. Connect the BT module to the mobile device.
7. Launch the Serial Bluetooth Terminal app.
8. Cover the black PMMA upper housing to power the top Qi receiver.
9. On the terminal app, “TF” and “F” are the target and the actual rotation frequency, respectively.
10. Type target rotation speed (Hz) in the Serial Bluetooth Terminal app, and APELLA will start rotating in a clockwise direction.
11. Cover the front P9 to block external light when performing LoD applications.
12. Type minus number (such as −10) to rotate in a counter-clockwise direction.
13. Type 77 in the terminal app to initiate a shaking sequence.
14. A more detailed operation video (APELLA operation.mp4) can be found in the OSF file repository.

## 7 Validation and characterization

### 7.1 Propeller driving mechanism characterization

**Fig. 7:**
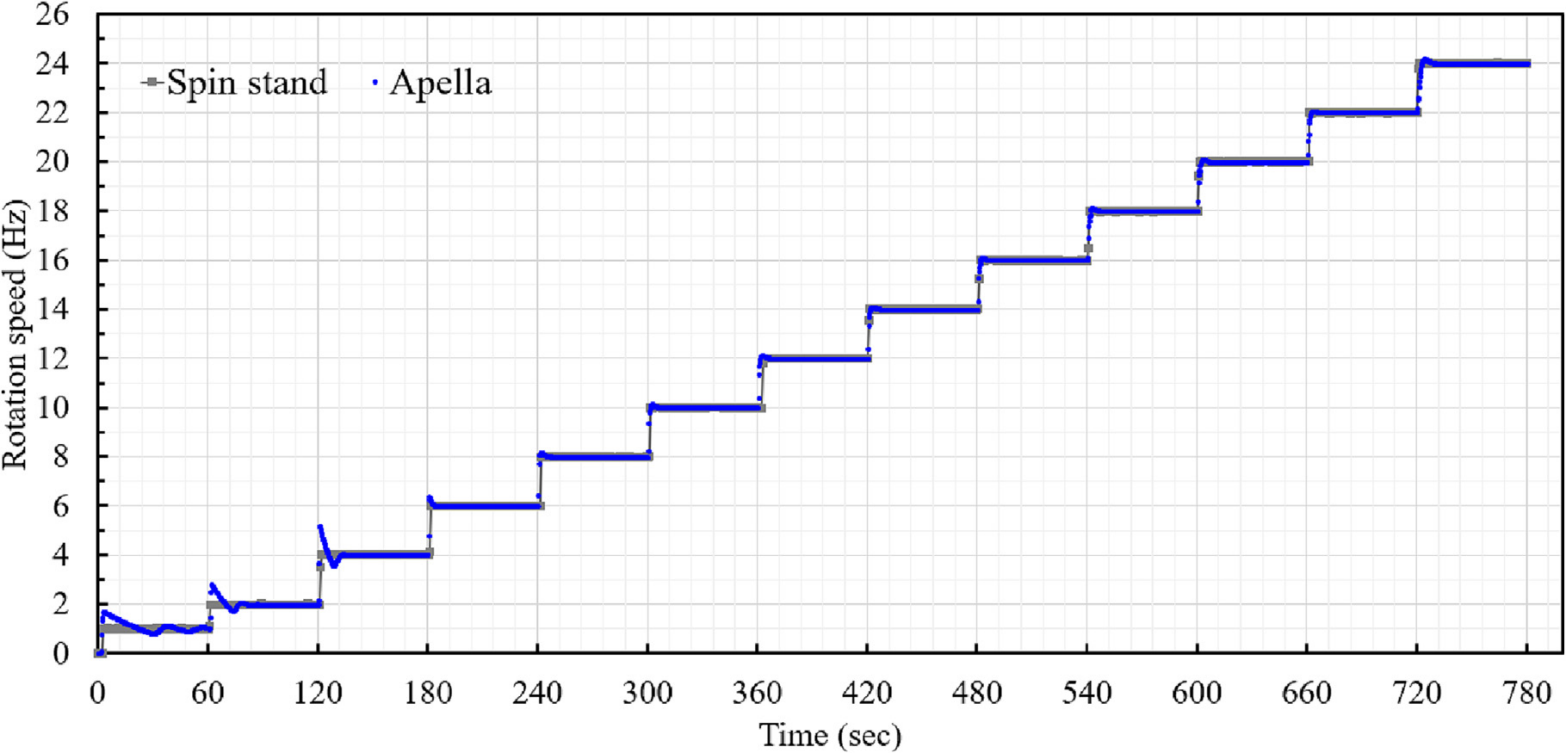
Speed comparison between conventional spin stand motor and the APELLA.

Fig. 7 illustrates a comparison of rotation speed control between a conventional spin stand and APELLA. The spin-stand motor displays perfect speed sequence steps. When APELLA starts to rotate from 0 Hz to 1 Hz, a higher speed (1.7 Hz) is used to overcome the rotational inertia. When increasing the rotation speed, there are large overshoots (29.5–70%) below 6 Hz and small overshoots (0.2–1.68%) above 6 Hz. APELLA has a resolution and a maximum rotation speed of 0.1 Hz and 24.5 Hz, respectively. APELLA has the capability to reverse the propeller rotation for deceleration, but its deceleration performance of 3.05 Hz/s is not optimal for siphon valves on LoD. However, since the rotation part has 5 V power source and an Arduino controller, it is possible to add an actuator for an “air break” feature similar to airplanes. There is room for APELLA to optimize PID parameters and improve aerodynamic design to reduce overshoot and increase the maximum speed.

### 7.2 Mixing efficiency between APELLA and conventional spin stand

**Fig. 8:**
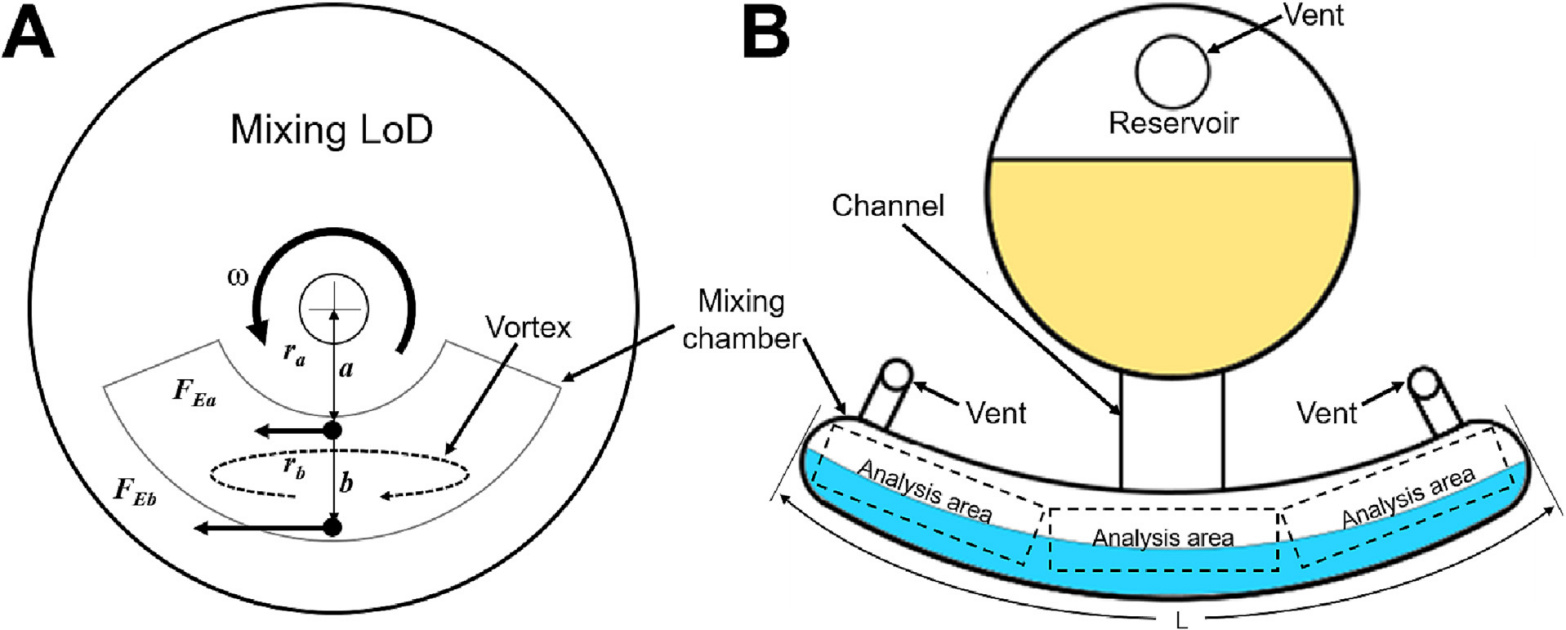
Euler force mixing principle and the mixing chamber design. **A)** The principle of Euler-force mixing. A vortex is generated by the difference in the Euler forces that agitates the solution inside a mixing chamber. **B)** A microfluidic mixing chamber for mixing using Euler force. A channel connects the reservoir and the mixing chamber. The channel has a width and a height of 2.5 mm and 0.5 mm, respectively. The reservoir and the mixing chamber have a depth of 2.17 mm, which are filled with the same volume of yellow and blue ink (Staedtler, Nürnberg, Germany) colored distilled water (5:100), respectively. Vents are used for introducing the liquid and releasing trapped air. Three different areas of the mixing chamber were analyzed for standard deviation calculation. (For interpretation of the references to color in this figure legend, the reader is referred to the web version of this article.)

Mixing solutions in a microliter scale volume with a low Reynolds number is challenging since turbulence is hard to achieve in these conditions. For LoD systems, an effective mixing method is based on the Euler force [25]. The Euler force is an inertial force that acts in a direction perpendicular to the radial direction of a disc. When the angular velocity of a rotating coordinate system is changed by acceleration or deceleration, the Euler force can be expressed by the following equation:

(1) $$F_{E}=m∙r\frac{d\omega }{\mathrm{dt}},$$

where $F_{E}$ is the Euler force (N), m is the mass in the rotating coordinate system (kg), r is the distance from the center of the rotating coordinate system to the mass (m), ω is the angular velocity (rad/s), and t is the time (s). Equation (1) shows that even at the same acceleration, the magnitude of *F_E_* differs depending on when r varies. Fig. 8A shows the principle of the Euler-force mixing while the disc rotates in a counter-clockwise direction. The Euler force $F_{\mathrm{Ea}}$ at point a is smaller than the force $F_{\mathrm{Eb}}$ at the point b since the distance to the center $r_{a}$ is smaller than the $r_{b}$. The difference in the Euler force generates a vortex that agitates the solution inside a mixing chamber. One can further enhance the mixing efficiency by changing the rotational speed and direction of the disc. Thus, we implement the Euler force mixing to evaluate APELLA.

The mixing efficiency quantitation is performed by analyzing the image inhomogeneity inside the mixing chamber [36]. The captured images are converted to an 8-bit grayscale by an image processing software, ImageJ [37]. The standard deviation, *s*, obtained by the inhomogeneity of the 3 different analysis areas in the mixing chamber is calculated from the luminance histogram as follows.

(2) $$s=\sqrt{\frac{1}{n}}\sum_{i=0}^{255}{\left(m_{i}-\mu \right)}^{2}f_{i}$$

(3) $$\mu =\frac{1}{n}\sum_{i=0}^{255}m_{i}f_{i}$$

(4) $$n=\sum_{i=0}^{255}f_{i}$$

Where $m_{i}$ is the gradation value and $f_{i}$ is the luminance of the gradation value. In this way, the standard deviation, *s*, can be used to describe the inhomogeneity of the solution inside the mixing chamber quantitatively. A good mixture of the solution correlates with a small standard deviation value.

The mixing LoD (Fig. 6) has four different arc-lengths (L = 10, 15, 20, and 25 mm) mixing chambers that are filled with four different volumes (40, 60, 80, and 100 μl) of yellow (inside the reservoir) and blue (inside the mixing chamber) solution for comparison of mixing efficiency. When the mixing LoD reaches a speed of 4 Hz, the meniscus at the interphase between the reservoir and the channel breaks, and the yellow solution flows into the blue solution contained in the mixing chamber. After this, the disc is shaken by changing the rotation direction every 180 degrees at a speed of 1 Hz for 160 s to engage the Euler force. We compared the mixing efficiency between the APELLA and a conventional spin stand (RE 35, Maxon motor AG, Sachseln, Switzerland) with the same visualization module (the rotational part of the APELLA was fixed on the spin stand), mixing LoD, and shaking parameters.

**Fig. 9:**
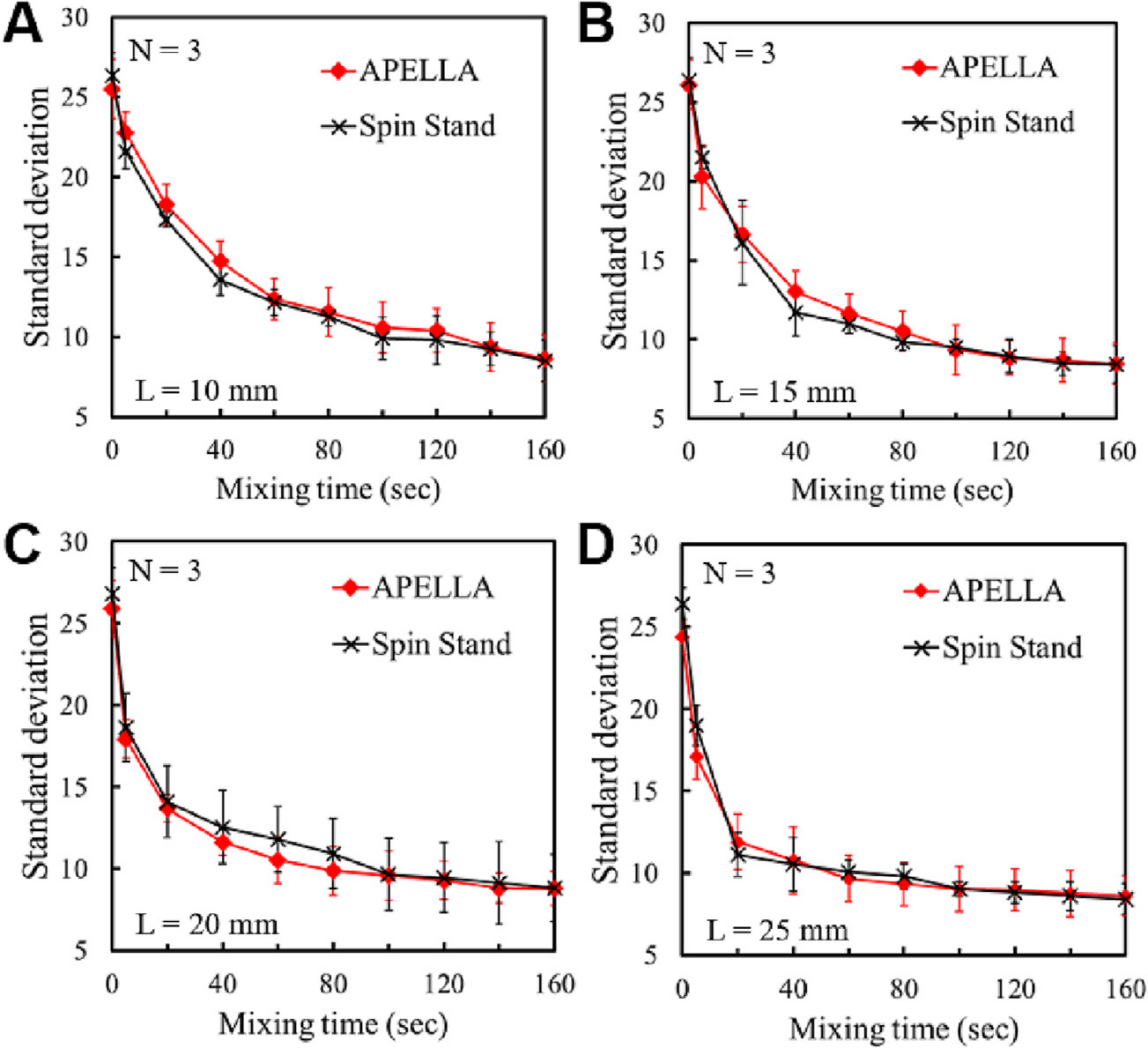
Comparison of mixing efficiency between APELLA and a conventional spin stand. **A**) L = 10 mm. **B)** L = 15 mm. **C)** L = 20 mm. **D)** L = 25 mm.

Fig. 9A-D demonstrate that the difference in overall mixing efficiency between the APELLA and the conventional spin stand is within the variance range. The experiment results show that the Euler force mixing performance of the APELLA is comparable to that of the conventional spin stand. The spin stand has a higher acceleration when changing the rotation direction. This explains why the 10 mm chamber mixing curve in Fig. 9A shows a lower standard deviation than the APELLA in the first 60 s. Additionally, the APELLA takes longer to reverse direction than the spin-stand due to its momentum.

Moreover, the mixing efficiency increases with the length of the mixing chamber, and a more considerable Reynolds number can explain this due to the higher solution volume. Thus the 25 mm mixing chamber reaches a standard deviation value close to 10 after 40 s compared to the 10 mm one, which reaches the same value but after 120 s.

### 7.3 High temporal resolution mixing visualization

The stroboscopic setup takes one image per disc revolution, which has a limited imaging temporal resolution at lower rotational speeds. However, in the case of LoDs, which prefer lower working speed [7] it is essential to have the temporal resolution independent from the rotation speed. To overcome this problem, the spy camera on the APELLA provides a field of view of 32 × 18 mm with HD (sensor resolution 1280 × 720 pixels) and high temporal resolution (30 frames per second) imaging, regardless of the rotational speed. Furthermore, the spy camera rotates with the disc increasing the image quality and reducing the complexity compared to the traditional stroboscopic method.

**Fig. 10:**
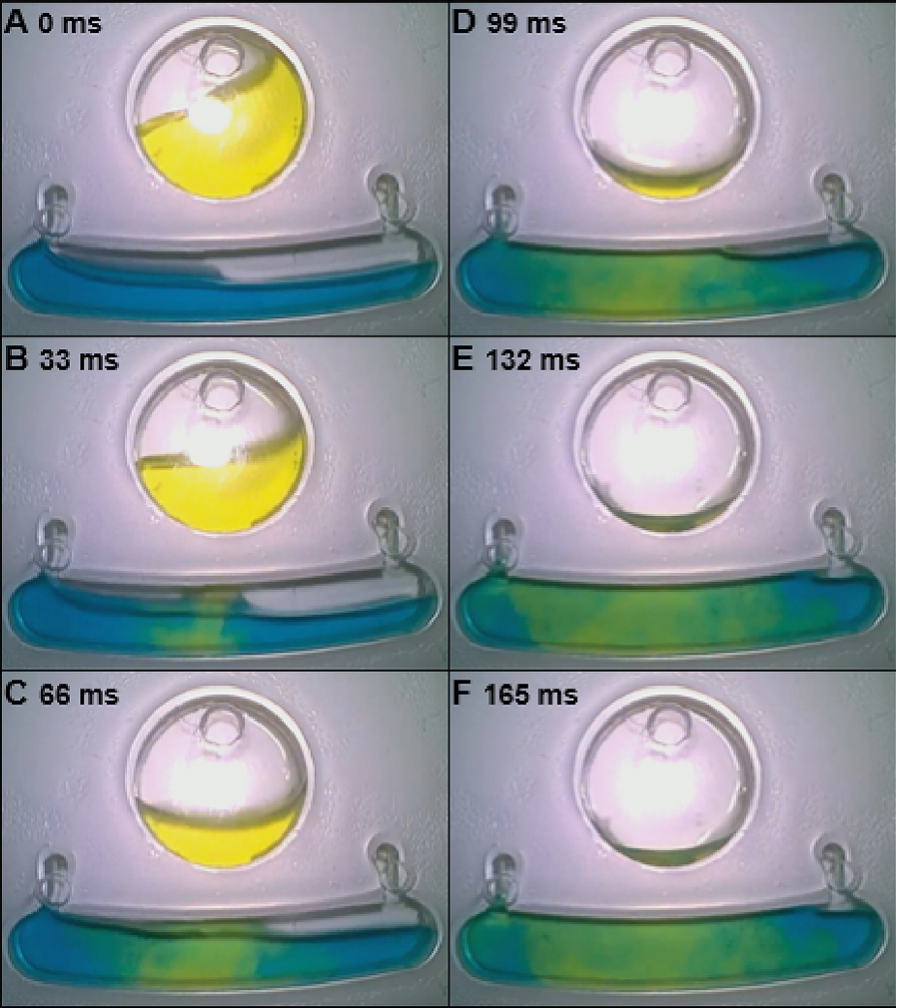
Mixing event (at a rotational speed of 5 Hz) captured by the spy camera with 33-millisecond intervals. **A)** Before the rapture of the meniscus at the interphase between the reservoir and the mixing chamber. **B)** Rapture of the meniscus and the two colors come in contact. **C)** Mixing flow created by the yellow solution. **D)** The yellow solution expanded in the mixing chamber. **E)** The yellow solution flow slowed down. **F)** End of the mixing created by the rapture of the meniscus and Euler force. (For interpretation of the references to color in this figure legend, the reader is referred to the web version of this article.)

The APELLA could capture the moment the yellow solution entered the mixing chamber (at 4 Hz speed), which is very difficult to capture with the stroboscopic method (imaging interval 200 ms) at the same speed. Fig. 10 shows six images of the mixing captured by the spy camera with an interval of 33 ms. While the yellow solution entered the mixing chamber, a turbulence flow was created simultaneously, as shown in Fig. 10B. From the spy camerás images, it was possible to calculate the velocity of the yellow solution during its expansion in the mixing chamber, which was found to be 41.7 mm/s. The microscale color pattern inside the mixing chamber changed rapidly and continuously. As the mixing time increased, the boundary between the two solutions became obscured, and the color of the liquid in the chamber became almost uniform until 200 s (without shaking). Unlike traditional stroboscopic setups, the spy camera on the APELLA can monitor only one mixing chamber at a time. Multiple spy camera modules are needed for simultaneous multi-chamber monitoring.

Table 1 highlights the parameters of APELLA in comparison to other LoD systems with imaging function.

**Table 1:** 

	Driving mechanism	Maximum speed (rpm)	Motor power source	Camera power source / operation time	Operation noise	System weight
Spin stand / Stroboscopic imaging [19]	Spindle motor	6,000	AC power supply, DC 24 V	AC 220 V / unlimited	Low	> 30 kg
Spin stand / co-rotating camera [25]	Spindle motor	1,800	AC power supply, N/A	Battery / limited	Low	N/A
Spin stand / co-rotating camera [21]	Spindle motor	5,000	Lab power supply, DC 24 V	Battery / Limited to 45 mins	Low	N/A
APELLA	Motor-propeller	1,440	Wireless / 5 V	Wireless 5 V / unlimited	High	590 g

In conclusion, the APELLA achieves:

• Unconventional propellor driving mechanism proof of concept.
• Low development cost with off-the-self consumer electronics.
• Closed loop and sequential speed control.
• Similar mixing efficiency compared to traditional spin stand.
• A high temporal resolution of mixing process imaging at a low rotational speed.
• Lightweight, portable size.
• Uses 6% power compared to traditional spin stand and stroboscopic system.

## Declaration of Competing Interest

The authors declare that they have no known competing financial interests or personal relationships that could have appeared to influence the work reported in this paper.
